# Structural Insights Into Key *Plasmodium* Proteases as Therapeutic Drug Targets

**DOI:** 10.3389/fmicb.2019.00394

**Published:** 2019-03-05

**Authors:** Manasi Mishra, Vigyasa Singh, Shailja Singh

**Affiliations:** ^1^Department of Life Sciences, School of Natural Sciences, Shiv Nadar University, Dadri, India; ^2^Special Centre for Molecular Medicine, Jawaharlal Nehru University, New Delhi, India

**Keywords:** malaria, proteases, papain-family cysteine proteases, aspartyl protease fold, Ca^2+^-dependent subtilase, therapeutics, drug targets

## Abstract

Malaria, caused by protozoan of genus *Plasmodium*, remains one of the highest mortality infectious diseases. Malaria parasites have a complex life cycle, easily adapt to their host’s immune system and have evolved with an arsenal of unique proteases which play crucial roles in proliferation and survival within the host cells. Owing to the existing knowledge of enzymatic mechanisms, 3D structures and active sites of proteases, they have been proven to be opportune for target based drug development. Here, we discuss in depth the crucial roles of essential proteases in *Plasmodium* life cycle and particularly focus on highlighting the atypical “structural signatures” of key parasite proteases which have been exploited for drug development. These features, on one hand aid parasites pathogenicity while on the other hand could be effective in designing targeted and very specific inhibitors for counteracting them. We conclude that *Plasmodium* proteases are suitable as multistage targets for designing novel drugs with new modes of action to combat malaria.

## Introduction: Parasite Life Cycle and the Role of Proteases

Malaria is a life-threatening parasitic disease transmitted to humans by the bite of an infected female *Anopheles* mosquitoes. Female *Anopheles* mosquitoes are the primary hosts and transmission vectors of malaria parasites while humans are the secondary hosts. It is caused by intracellular apicomplexan parasite *Plasmodium*. Principally, four species of the genus *Plasmodium* infect humans: *P. ovale*, *P. malariae*, *P. vivax*, and *P. falciparum*. *P. falciparum* is the most prevalent species of malaria in humans and responsible for 80% of all malaria infections and severe complications leading to death. Several classes of drugs like, quinolines (chloroquine, quinine, mefloquine, amodiaquine), antifolates (pyrimethamine, chloroguanide proguanil, paludrine, sulfadoxine, sulfalene, dapsone) are vital part of antimalarial chemotherapy (Cunha-Rodrigues et al., 2006; Nigussie et al., 2015). Artemisinin, a sesquiterpene lactone isolated from plant *Artemisia annua* is another popular antimalarial drug which shows activity against all parasite stages with in erythrocytes (Klayman, 1985). Artemisinin based compounds used in combination with drugs with different mode of action (artemisinin-combination therapies, ACT) are now the standard and most recommended treatment for malaria worldwide. Most of the anti-malarial drugs target major metabolic pathways such as nucleic acid metabolism, Hb metabolism, heme detoxification, mitochondrial electron transport, oxidative stress, fatty acid and phospholipid biosynthesis, and transport proteins at the host pathogen interface. However, the emergence of resistance to most of the antimalarial drugs including ACTs have been widely reported (Alam et al., 2009). Therefore, identification of novel multistage targets and new modes of action is essential to develop new drugs against resistant malarial parasites.

**FIGURE 1:**
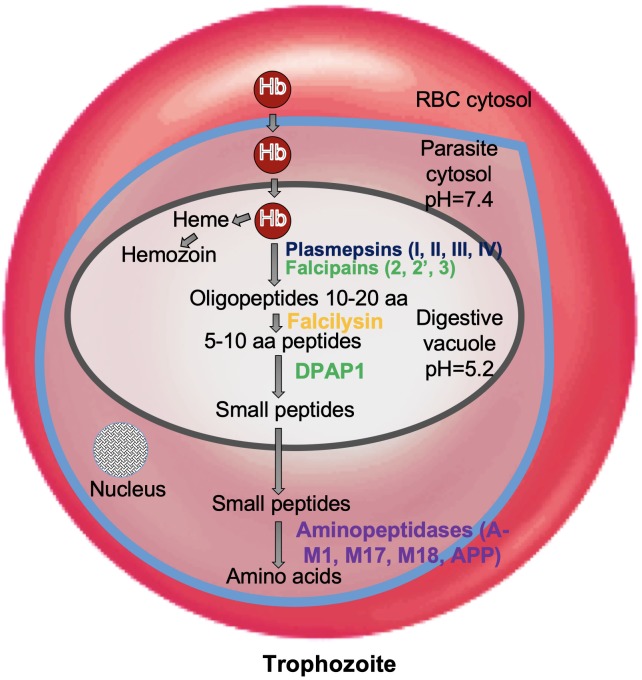
Role of proteases in hemoglobin degradation. Host Hb degradation takes place within the digestive vacuole primarily by co-ordinated action of plasmepsins and falcipains. Small peptides are further converted into amino acids by aminopeptidases. Amino acids are transported to parasite cytosol by an ATP-dependent membrane transporter.

*Plasmodium falciparum* undergoes a complex life cycle within human host and the mosquito vector. Malaria infection is initiated in human host by transmission of sporozoites from the infected mosquito. These sporozoites establish an asymptomatic infection in hepatocytes and multiply asexually to form several merozoites which are released in to the blood stream. Merozoites actively invade the RBCs and this marks the start of 48 h erythrocytic cycle. Erythrocytic cycle consists of RBC invasion, Hb degradation, schizont rupture, release of merozoites and re-invasion in to non-infected erythrocytes. Inside RBC, the parasitophorus vacuole (PV) and its membrane (PVM) surrounds the developing parasite. Parasites develop from small “ring-stage” to larger active “trophozoites,” and then to multinucleated “schizonts.” During this metamorphosis, malaria parasites use Hb degradation as a source of free amino acid to fulfill their nutritional demands for growth and maturation. Trophozoites ingest erythrocyte cytoplasm and transport it to large central DV which is analogous to lysosomes. In this pathway, several proteolytic enzymes are known to be involved: aspartic proteases (Plasmepsins I, II, III, IV), three papain-family cysteine proteases (Falcipain-2, 2′ and 3), metalloprotease (Falcilysin), and dipeptidyl aminopeptidase 1 (DPAP1) (McKerrow et al., 1993; Gluzman et al., 1994; Blackman, 2000; Rosenthal, 2004, 2011; Klemba and Goldberg, 2012; Figure 1). In DV, Hb is broken down to smaller peptides and transported to the parasite cytosol for converting in to free amino acids by exopeptidases known as neutral aminopeptidases (PfA-M1, PfA-M17, PfA-M18, Pf-APP) (Dalal and Klemba, 2007; Teuscher et al., 2007). These free amino acids are utilized by the parasite for protein synthesis and various metabolic pathways leading to metamorphosis. Erythrocytic cycle is brought to an end at the rupture of erythrocytes by mature schizonts thereby releasing the invasive merozoites which invade fresh RBCs. These repeated cycles of asexual multiplication and toxic material from infected RBCs are responsible for clinical symptoms of the disease.

It has been well recognized that *Plasmodium* frequently express different types of protease activities at different stages of its life cycle to support parasite replication and metamorphosis (McKerrow et al., 1993; Blackman, 2008). In this review, we discuss the crucial biological roles of essential proteases during *P. falciparum* infection, with an emphasis on their peculiar structural features projecting them as promising drug targets for malaria chemotherapy.

## Subtilisin-Like Proteases: Structural Evolution Towards Specific Ca^2+^-Dependent Regulation

**FIGURE 2:**
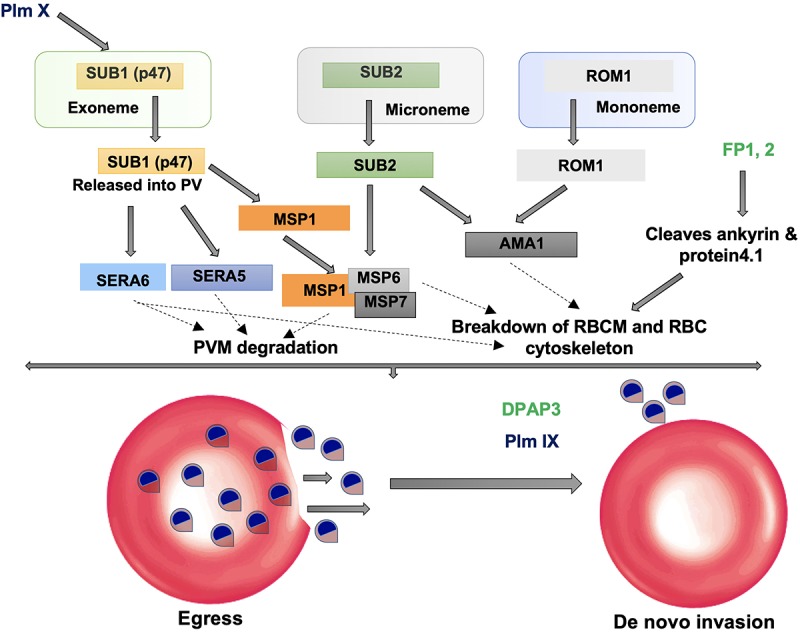
Role of proteases in invasion and egress. Subtilisin-like proteases (SUB1 and SUB2) are mainly involved in the egress and invasion process. SERA5 is involved in the destabilization of the PV through degradation of its membrane proteins and also involved in merozoite priming through regulation of erythrocyte binding ligand MSP. ROM1 catalyzes the intramembrane cleavage of various merozoite adhesins such as AMA1 and helps in invasion process.

Subtilisin-like proteases namely, PfSUB1, 2, and 3 are highly expressed proteases at late asexual blood stages of *Plasmodium* life cycle and are mainly involved in invasion and egress during parasitic asexual blood stage (Yeoh et al., 2007; Arastu-Kapur et al., 2008). SUB1 executes the processing of proteins involved in egress of merozoites from host hepatocytes or erythrocytes and SUB2 is required for processing of proteins involved in invasion of erythrocytes by merozoites. PfSUB1 is mainly expressed during the late stages of liver schizont maturation and is necessary for the parasite to successfully complete liver phase and for intracellular growth/development of the parasite before egress (Blackman et al., 1998; Pino et al., 2012; Suarez et al., 2013). Invasion of erythrocytes by merozoites as well as the rupture of erythrocytes by schizonts, i.e., egress are complex processes involving hydrolysis of erythrocyte cytoskeletal proteins and proteolytic processing of several parasite proteins. Egress from infected erythrocytes is a two-step process: disruption of PVM followed by erythrocyte membrane (RBCM) (Figure 2). Mature PfSUB1 after a series of proteolytic processing events, is stored in specialized secretory apical organelles of merozoites called exonemes, and discharged in to the PV on rise of intracellular Ca^2+^ during final stages of schizont maturation initiating the egress (Yeoh et al., 2007; Collins et al., 2013; Figure 2). Serine repeat antigen family proteins (SERA5 and SERA6) and merozoite surface protein 1 (MSP1) being processed by mature PfSUB1, play crucial role in destabilization of RBC cytoskeleton (Ruecker et al., 2012; Das et al., 2015). SUB1 is also known to carry out the processing of MSP1 complex and cleave a number of rhoptry proteins which are important for invasion. *Plasmodium* rhomboids and SUB2 are involved in shedding the protein coat of the merozoite and crucial for parasite attachment to the RBC surface during the invasion process (Baker et al., 2006; Figure 2).

**FIGURE 3:**
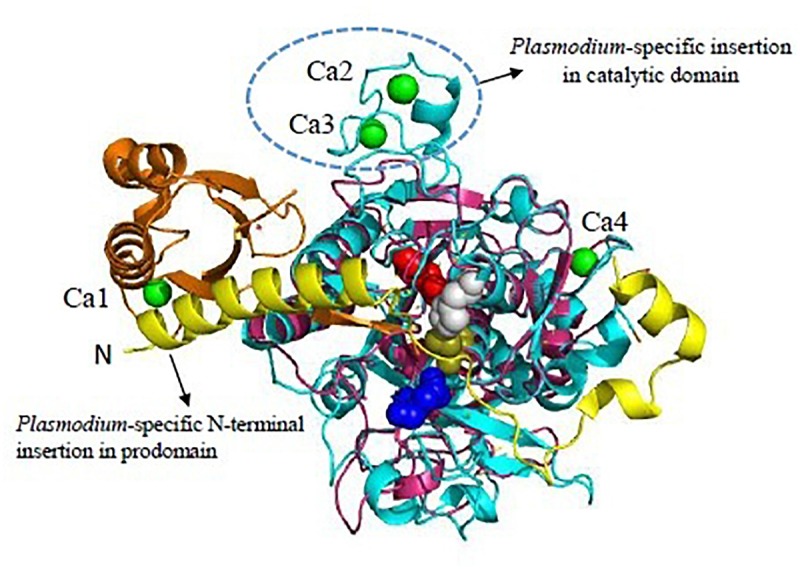
Unique features of *Plasmodium vivax* SUB1 structure. Cartoon representation of overall structure of PvSUB1 (PDB: 4TR2). Overall structure comprises N-terminal insertion in prodomain comprising the “belt” domain (shown in yellow) followed by a classical bacterial-like prodomain (orange), and the C-terminal subtilisin-like catalytic domain (cyan). The catalytic triad (Asp316/His372/Ser549; Red/Gray/olive) is shown as spheres. The oxyanion hole residue (Asn464) is shown as blue sphere. Bound calcium ions are shown as green spheres. Out of four calcium binding sites, three (Ca1, Ca2, Ca3) are specific to parasite subtilases. Ca4 is conserved in all homologs of subtilisin. *Bacillus* subtilsin (PDB: 1SUP) is superimposed (shown in magenta) highlighting the unique insertions in parasite subtilases. All structure figures have been made in Pymol.

SUB1 is highly conserved across all *Plasmodium* species sharing 30% sequence identity in the catalytic domain. Overall structure consists of two distinct structural domains: An N-terminal prodomain region which is tightly bound to the catalytic domain. The *Plasmodium* SUB1 prodomain has a striking difference from prodomains of other known subtilases. A structurally well-defined N-terminal insertion in the prodomain forming a conserved α-helix which is followed by a long connecting loop that folds like a “belt” over the enzyme active site was revealed by the crystal structure of PvSUB1 (Giganti et al., 2014; Figure 3). Presence of the “belt” domain in all *Plasmodium* SUB1 sequences had suggested its important functional role most likely in stabilizing SUB1 after prodomain cleavage and in regulation of SUB1 activity. First crystallographic structure of PfSUB1 at 2.25 Å in complex with its prodomain highlighted the calcium dependence of SUB1 (Withers-Martinez et al., 2014). The prodomain displays a Ca^2+^ binding site which is conserved in all *Plasmodium* orthologs but absent in bacterial subtilases (Ca1 in Figure 3). The catalytic triad (Asp316, His372, and Ser549) is highly conserved among different SUB1 orthologs having variations only in peripheral loops. However, a *Plasmodium*-specific insertion of ∼25 residues has been found in PvSUB1 and also PfSUB1 which is absent in bacterial homologs (Suarez et al., 2013; Withers-Martinez et al., 2014). This region includes a conserved sequence motif (E-X7-DDD-X-N-X3-DD) and defines a metal-binding site that accommodates two Ca^2+^ ions (Ca2 and Ca3 in Figure 3). In addition to this, PvSUB1 catalytic domain contains a third Ca^2+^ binding site which is conserved in all bacterial homologs (Ca4 in Figure 3). Further PvSUB1 catalytic domain is stabilized by three disulfide bridges which are conserved in all *Plasmodium* SUB1s. Crystal structures have also revealed the unusual interaction of SUB1 active site residues with substrate residues on both prime and non-prime sides of the scissile bond (Withers-Martinez et al., 2014). These unique structural features of *Plasmodium* subtilases suggests that parasites have evolved a specific Ca^2+^-dependent regulation of SUB1 activation to initiate the merozoite egress from the host cells (Withers-Martinez et al., 2014). The *Plasmodium* specific structural insertions in the prodomain and the catalytic domain of SUB1 are critical for its activity, define its substrate specificity and also essential for parasite development *in vivo*. Therefore, *Plasmodium* subtilases are appealing targets for development of novel anti-malarials.

Mature PfSUB2 is secreted into micronemes of the merozoites and helps in invasion of RBCs by merozoites and also regulates the sheddase activity which involves release of MSPs and others during the erythrocyte invasion (Harris et al., 2005; Silmon de Monerri et al., 2011). Therefore, events during invasion and egress at erythrocytic and pre-erythrocytic stages are tightly regulated by activity of SUB proteases and offer two critical checkpoints where parasite development can be blocked. Owing to the dual role of SUB1 in hepatic as well as erythrocytic phases, it has been an attractive multi-stage target for antimalarial drug development (Suarez et al., 2013). In various studies, low molecular weight compounds and natural compounds effectively blocking PfSUB1 mediated egress and inhibiting erythrocyte invasion by merozoites have been identified (Yeoh et al., 2007; Calderon et al., 2011; Moneriz et al., 2011; McConville et al., 2015). “Pan-reactive” drug-like compounds such as peptidyl alpha-ketoamides have been developed based on the unusual characteristics of substrate binding cleft of SUB1. These protease inhibitors could effectively block SUB1 orthologs from *P. falciparum*, *P. knowlesi*, *P. vivax*, and *P. berghei* (Withers-Martinez et al., 2012). In another high-throughput screening of 450 peptidic and non-peptidic compounds, various synthetic hydrazones and hydrazides such as quinolylhydrazone 2 have been identified which showed potent inhibitory properties against PfSUB1 (Gemma et al., 2012). Their inhibitory effect led to enrichment of unprocessed MSPs on surface of merozoites that significantly inhibited erythrocyte invasion. Apart from these drugs, inhibitory regions of the prodomains have been also targeted for development of peptidomimetic inhibitors against PfSUB1 and PfSUB2 (Yabuta et al., 2001; Fugère and Day, 2002; Harris et al., 2005). A series of difluorostatone-based inhibitors of PfSUB1 have been analyzed using homology modeling and molecular docking approaches in lieu of development of potent “pan”-SUB1 inhibitors (Brogi et al., 2016). In recent approaches, computational analysis together with *in vitro* testing have been useful in rationalizing the structural requirements for inhibition of SUB1 orthologs.

## Malaria Parasite Plasmepsins: Distinct From Other Aspartyl Proteases

*Plasmodium falciparum* genome has revealed the presence of at least ten genes encoding aspartic proteases namely plasmepsins (Plm) I, II, III, IV, V–X and a HAP. Genes for Plms I, II, IV and HAP cluster on chromosome 14 and share more similarity (60–70%) with each other as compared to Plms V–X. Studies have suggested expression of Plm I, II, IV, V, IX, X and HAP in the erythrocytic stage whereas Plm VI, VII, and VIII being expressed in exo-erythrocytic stage (Banerjee et al., 2002). Plms I, II, and IV belong to A1 family aspartic proteases having dual aspartate active site configuration, whereas HAP, contains a histidine in place of one of the catalytic aspartic acid residues (Klemba and Goldberg, 2005). Like all aspartic proteases, Plms involve a catalytic water molecule and generate a “tetrahedral intermediate” while acting upon their substrates. Plms along with cysteine proteases and metalloproteases function in acidic food vacuole to participate in orderly Hb degradation pathway (Figure 1). Plm I and Plm II are 75% identical in sequence and cleave the Phe33-Leu34 peptide bond of Hb which is considered to be the critical first step in the degradation pathway. Plm I readily cleaves native Hb and Plm II has a preference for acid-denatured globin (Gluzman et al., 1994).

**FIGURE 4:**
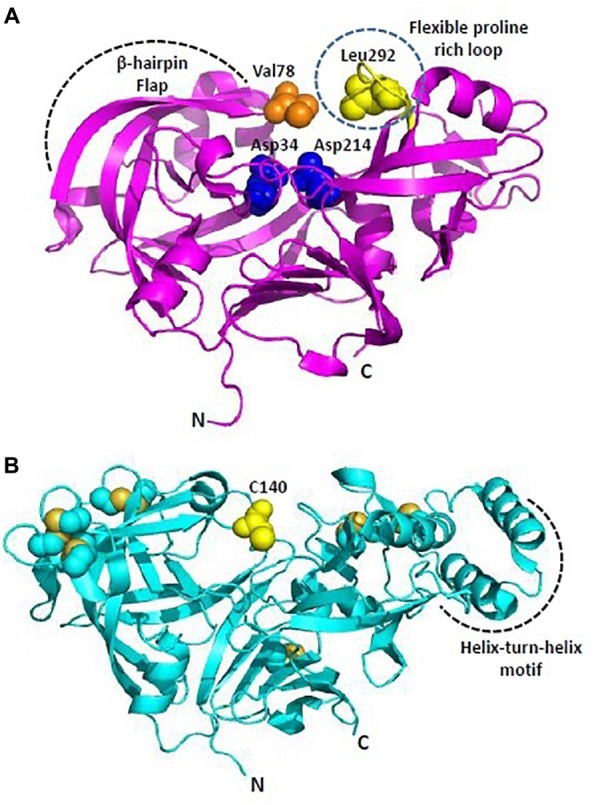
Overall structure of *Plasmodium* plasmepsins. **(A)** Cartoon representation of overall structure of Plasmepsin II (PDB: 1IF4). Flexible proline rich loop is depicted in yellow and catalytic aspartates Asp34 and Asp214 are shown as blue spheres. Residues defining the catalytic cavity Val78 (orange) and Leu292 (yellow) are shown as spheres. **(B)** Cartoon representation of overall structure of Plasmepsin V (PDB: 4ZL4). Free C140 (within the flap) and cysteine residues forming disulfide linkages are shown in yellow. Helix-turn-helix motif is unique to PlmV unlike all other plasmodial plasmepsins and aspartyl proteases. All structure figures have been made in Pymol.

Among Plms, Plm II emerged as a target for drug intervention because its crystal structure in complex with pepstatin A was reported quite early in 1996 (Silva et al., 1996). Later, crystal structures of uncomplexed Plm II and complexed Plm II with potent inhibitors were solved up to high resolution limits of 1.9 and 2.7 Å (Asojo et al., 2003). Mature Plm II consists of a single chain of 329 amino acids, folded in to two topologically similar N and C terminal domains (Asojo et al., 2003; Dan and Bhakat, 2015). Each domain contributes one aspartic acid residue to the catalytic dyad (Asp34 and Asp214) defining the active site (Figure 4A). N-terminal domain forms a distinct β-hairpin structure, known as “flap” which covers the binding cavity and is able to interact with substrates and inhibitors. The relative opening of flap upon inhibitor uptake, further stabilization by hydrogen bond interactions and the flexible proline rich loop (Ile290-Pro297) are responsible for uncomplexed and complexed conformations of Plm II in order to hold the inhibitor in its active site. The extent of flap opening in order to accommodate incoming inhibitor is estimated by measuring distance between C-α residues of Val78 and Leu292 (Figure 4A). Bound and open conformations of PlmII have suggested that in coordination with the “flap” region and the proline rich flexible loop it can accommodate inhibitors of varying sizes. This huge conformational flexibility at the binding cavity has played rather important role in Plm II inhibitor design strategies. Generally, transition-state analogs of the tetrahedral intermediate which bind more tightly as compared to the substrate but are resistant to enzymatic cleavage are popular as aspartic protease inhibitors. Thus, several peptidomimetics acting as transition-state isostere, based on reduced amide, statin, hydroxyethylamine scaffolds, and also small molecule inhibitors of Plm II have emerged as potential antimalarial agents (Boss et al., 2003; Ersmark et al., 2006; Dan and Bhakat, 2015). However, the major bottleneck has been the specific selectivity to pathogenic Plms and moreover selectivity toward a particular subtype of Plm. Recently, structure-guided optimization of compounds based on hydroxyethylamine core scaffold have led to development of potent plasmepsin inhibitors (Plm I, II, and IV) also displaying nanomolar activity in cell based models (Jaudzems et al., 2014; Pino et al., 2017). 1*S*R, is a potent Plm IV inhibitor (IC_50_ = 29 nM) but is not a selective inhibitor of Plms over the human aspartic protease cathepsin D (Jaudzems et al., 2014). *C*2 symmetric hydroxyethylamine analogs (10f, 10g, 12c) have also been reported as potent inhibitors of Plm II and Plm IV and showed significant growth inhibition of *P. falciparum* in *in vitro* cultures (Singh et al., 2018). Using the structure-guided drug discovery, a series of compounds based on alkylamino scaffold with nanomolar inhibition against Plm II have been recently developed (Mishra et al., 2018). These compounds (KNI-10742, 10743, 10395, 10333, 10343) also show significant antimalarial activity and thus could be promising candidates for further drug development.

Plm V plays crucial role in recognizing and processing effector proteins for export to host cells (Boddey et al., 2010; Russo et al., 2010). Plm V recognizes and cleaves a pentameric sequence (RxLxE-Q-D) known as PEXEL motif, uncovering the export signal (xE-Q-D) and translocating the proteins to parasite-infected erythrocytes. The P_1_ leucine in the PEXEL is known to be crucial for binding of Plm V substrates and inhibitors. Therefore, inhibitor design strategies have primarily focused on mimicking the transition state PEXEL. PEXEL-mimicking small molecule inhibitors of Plm V have shown lethal inhibition of *P. falciparum* growth indicating its crucial role in protein export and importance of this process in parasite’s survival. WEHI-842, a potent inhibitor of Plm V (IC_50_ ∼0.2 nM) mimics the transition state of amide bond proteolysis for PEXEL substrates, efficiently blocks protein export and inhibits parasite growth (Hodder et al., 2015). High resolution crystal structure of *P. vivax* Plm V-WEHI-842 revealed a typical aspartyl protease fold (N and C-terminal subdomains anchored via six-stranded interdomain β-sheet) with a single hairpin loop as “flap” over the substrate binding pocket which accommodates the inhibitor WEHI-842. In WEHI-842, P3 arginine is replaced by proteinogenic amino acid, canvanine and mimics the RVL amino acid sequence of PEXEL. The study of enzyme-inhibitor complex has revealed that the substrate binding pocket can be better utilized or efficiently filled by non-peptide based inhibitors leading to improved affinities for Plm V (Hodder et al., 2015). Disulfide bond architecture in *P. vivax* Plm V was found to be more complex than other members of the aspartyl protease family. The mature domain of *P. vivax* Plm V contained 15 cysteine residues forming seven disulfide linkages: four located in the N-terminal subdomain and three in the C-terminal subdomain (Figure 4B). Flap sequence contains an unpaired C140, which is highly conserved in Plm V orthologs in *Plasmodium* species. Plm V shares this distinct structural feature with few plant and fungal aspartic proteases. Helix-turn-helix motif of *P. vivax* Plm V is another unique feature distinct from other Plms (Figure 4B). The hydrophilic residues lining the helix-turn-helix motif are highly conserved in Plm V orthologs across *Plasmodium* species and indicate important functional roles of this structural element. Therefore, given its importance in the parasite’s survival and also relative evolutionary distance from other mammalian aspartyl proteases, Plm V has gained attention as a potential drug target for development of new antimalarials. Though, several naphthoquinone derivatives and PEXEL-based peptidomimetic compounds inhibiting Plm V activity have been identified, yet they require further chemical modifications and research to establish their antimalarial potential as a drug (Sittikul et al., 2018). PEXEL peptidomimetic analogs with either cyclohexylglycine or phenylglycine in the P_2_ position have been identified as most potent inhibitors of Plm V till date (Nguyen et al., 2018).

Plm IX and X are known to be expressed in mature blood-stage schizonts and invasive merozoites. There functions had remained unknown, until recent studies identifying their essential roles in invasion and egress (Nasamu et al., 2017; Pino et al., 2017). Using conditional knockdown technology, Nasamu et al. (2017) have shown that Plm IX is essential for erythrocyte invasion, localizing to the rhoptries and acting as a maturase for proteins in this organelle while Plm X is crucial for both egress and invasion, controlling maturation of SUB1 in exoneme secretory vesicles. Plm X is important for final SUB1 processing step and appears to be the most upstream protease in the egress cascade. A pleiotropic hydroxyethylamine scaffold based Plm inhibitor (compound 49c) shows inhibition of Plm IX and X, blocking the blood-stage parasite egress and invasion as well as hepatic-stage egress and transmission (Pino et al., 2017). Compound 49c is a multistage peptidomimetic competitive inhibitor known to be effective against *P. falciparum in vitro* and the rodent parasite *P. berghei in vivo* (Guiguemde et al., 2010; Ciana et al., 2013). These recent reports confirm the suitability of Plm IX and X as plausible targets for further development of antimalarials.

It is evident that several Plms are essential for parasite survival and have been popular as drug targets. However, Plms share varying sequence homology with human aspartic proteases viz., lysosomal enzyme cathepsin D, cathepsin E, renin and pepsin A. Specifically, Cat D and Cat E are relevant because of their cellular location. They are easily accessible to the orally administered drugs targeting vacuolar aspartic proteases of *Plasmodium* (Coombs et al., 2001). Plm II and Cat D share 35% sequence identity and crystal structures show more or less similar active sites and similar binding with pepstatin A (Ersmark et al., 2006). Therefore, designing of inhibitors targeting Plms and not interacting significantly with human counterparts still remains a difficult challenge. In a GlaxoSmithKline (GSK) phenotypic screening of hydroxyethylamine based Plm inhibitors, potent anti-malarials with up to 50-fold Plm IV/CatD selectivity factor have been developed after optimizing the modifications in the selectivity-inducing structural motifs (Zogota et al., 2018). The mechanism of action of these compounds have been also linked to inhibition of Plm X.

## Falcipains, Unique Cysteine Proteases With Hemoglobin Binding Hairpin

Cysteine proteases are enzymes which share a common catalytic mechanism involving a nucleophilic cysteine thiol in their catalytic triad. The best characterized cysteine proteases of *Plasmodium* belong to papain-family (Clan CA, family C1) which are characterized by the presence of conserved Cys, His, and Asn in their catalytic triad. Genome analysis has revealed presence of four falcipains (FPs-1, 2, 2′, and 3), three dipeptidyl peptidases (DPAP-1, -2, -3), nine SERA proteins and a homolog of calpain (Wu et al., 2003; Rosenthal, 2011). Expression level of these proteases differ depending on the parasitic stage and they are involved in wide range of biological processes including degradation of Hb, protein trafficking, erythrocyte membrane rupture, *de novo* invasion of erythrocytes and egress from host erythrocytes and hepatocytes (Rosenthal, 2004; Rosenthal, 2011). FPs, majorly FP-2 and FP-3 are principal food vacuolar hemoglobinases which rapidly cleave Hb at multiple sites leading to its efficient degradation. FP-2 is also involved in degradation of erythrocyte-membrane skeletal proteins including ankyrin and band 4.1 protein (Hanspal et al., 2002). FP-1 plays a role in host cell invasion and might also be involved in oocyst production within the anopheles mosquito vector (Greenbaum et al., 2002; Eksi et al., 2004).

**FIGURE 5:**
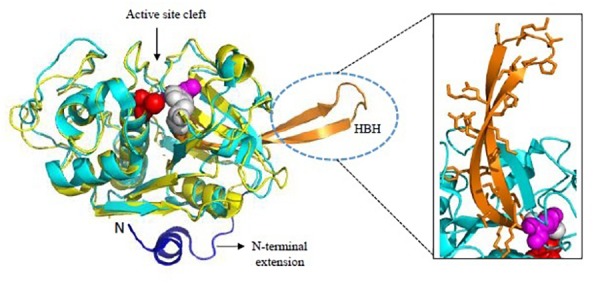
Overall structure of Falcipains. Cartoon representation of Falcipain-2 (PDB: 2GHU) displaying two distinct domains and a prominent active site cleft. Active site residues Cys-42 (Red), His-174 (Gray), and Asn-204 (Magenta) are shown as spheres. Papain (PDB: 9PAP; shown in yellow) as a model papain-like cysteine protease is superimposed. N-terminal extension (shown in blue) and the hemoglobin binding hairpin (HBH- shown in orange) are specific to plasmodial falcipains and not observed in other papain-like cysteine proteases. A detailed view of side chains of residues in the HBH are shown in the inset. All structure figures have been made in Pymol.

FP-2 and FP-3 are homologous to each other with 68% sequence identity between their catalytic domains while FP-1 shares less similarity (only 38% with FP-2 and FP-3). Crystal structure of mature FP-2 at 3.1 Å resolution revealing its novel structural features had been reported by Hogg et al. (2006) whereas first crystal structures of FP-2 (2.9 Å) and FP-3 (2.5 Å) in complex with small molecule inhibitors were reported later in 2009 (Kerr et al., 2009). FPs possess some unique structural domains as compared to other papain-like cysteine proteases. The N-terminal part of the prodomain functions in trafficking of FPs to the food vacuole and the C-terminal part of prodomain acts as an inhibitor of the mature domain, like other cysteine proteases of the C1 family (Pandey, 2011). However, FPs possess short N-terminal extensions (about 12 aa) of the mature domain which is critical for folding of the mature domain to active enzyme (Figure 5). A deletion construct of these 12 aa N-terminal segments was incapable of correct folding indicating that though short but it is significant element of secondary structure (Sijwali et al., 2002). The carboxy terminus possess a unique motif, an anti-parallel β-hairpin between the highly conserved active site histidine and asparagine residues, called Hb binding domain (Figure 5, inset). Such a motif of 14 aa is found in all homologs of falcipain though with sequence variations. The size of this motif varies in SERA proteases (10 aa) and DPAP I (8 aa). The specific role of this motif in capturing Hb has been validated by several studies (Pandey et al., 2005; Subramanian et al., 2009). The binding of Hb to FP-2 is strictly pH dependent. FPs have eight cysteine residues forming four disulfide bonds (Cys39-Cys80, Cys73-Cys114, Cys99-Cys119, and Cys168-Cys229). Cys99-Cys119 is unique to FPs and its parasite homologs whereas other three are well conserved among the papain-like enzyme structures (Hogg et al., 2006). Mutations in Cys99 and/or Cys119 have resulted in disrupted protein folding and inactive conformation of falcipain.

Inhibitors of falcipain block Hb hydrolysis and thus disrupt the parasite development. Serious efforts have been made for targeting FPs through two different approaches: (i) peptide/peptidomimetic covalent inhibitors and (ii) non-peptidic small molecule inhibitors. Covalent inhibitors like peptidyl vinyl sulfones, furanone derivatives, epoxy-succinate and fluoromethyl ketones (FMKs), various pyrimidine nitrile derivatives such as peptidic 2, 4-substituted pyrimidine nitriles and some purine nitriles are reported to have inhibitory activity against cysteine proteases at a nanomolar range blocking parasites’ ability of Hb hydrolysis leading to Hb-filled DVs that hampers the development of trophozoites (Olson et al., 1999; Lee et al., 2003; Ettari et al., 2010). However, many limitations have been faced with peptide based inhibitors such as lower specificity for malarial cysteine proteases in comparison to host ones, easily targeted by host proteases, poorly absorbed through cell membranes and sometimes cytotoxic properties (Shah et al., 2011; Chakka et al., 2015). Natural cysteine protease inhibitors such as Leupeptin, E-64 and Chymostatin also led to accumulation of undigested globin in the DV and resulting in to distorted morphology of the parasite at the trophozoite stage (Rosenthal, 1995; Semenov et al., 1998). Various non-peptidic small molecule inhibitors of FPs have been developed based on structure-based virtual screening of 3D models. Desai et al. (2004) identified various non-peptidyl cysteine protease inhibitors through virtual screening of ChemBridge database (containing approx. 2,41,000 compounds). Similarly, Li et al. (2009) identified various structurally different and effective non-peptidyl inhibitors through screening of SPECS database. Use of natural product based inhibitors as antimalarials has also gained positive reception in recent times due to their reduced cytotoxicity effects and higher availability. Gallinamide A/Symplostatin 4 and their synthetic derivatives can potentially inhibit FPs at low- to mid-nanomolar concentrations in the DV of parasites without showing any cytotoxic effects against human cell lines or erythrocyte lysis even at a very high concentration (>25 μM) (Stolze et al., 2012). Other chemotypes been reported as active inhibitors of FP-2 include thiosemicarbazones, sulfadoxine, semicarbazones, isoquinolines, pyrimethamine, chalcones, cycloguanil, mefloquine, and benzimidazole acrylonitriles (Singh and Rosenthal, 2001; Biot et al., 2007; Bhattacharya et al., 2009; Sharma et al., 2018).

Most of these reported inhibitors are directed toward clan CA proteases and are not specific for a particular protease within this large clan. The active site cleft represents the prime target for therapeutic intervention of clan CA proteases. This confers to the low selectivity of many of the active site FP-2 inhibitors against the human cysteine proteases (Cathepsins B, K, L, and S). A series of *P2*-modified aminonitrile based inhibitors of FP-2 have been designed with rather limited selectivity toward off-target proteases of cathepsin family (Nizi et al., 2018). However, the essential Hb binding motif observed only in falcipain subfamily points toward the need for design of peptidomimetic drugs that could disrupt the critical interaction of falcipain with its target, i.e., Hb. Moreover, recent reports on design, synthesis and effects of azapeptides as allosteric inhibitors of FPs suggests alternative modes of inhibition that can be focused on. These azapeptide compounds interact with the enzymes in presence of their prodomains and block their auto-processing in to mature FPs thereby arresting the parasite growth (Pant et al., 2018). It is advocated that targeting the allosteric site could be a better alternative than targeting the active site in terms of high selectivity and low vulnerability to drug resistance.

Dipeptidyl aminopeptidases are papain-fold cysteine proteases that cleave dipeptides from the N-terminus of protein substrates. Using vinyl sulfone inhibitor SAK1, it had been shown that DPAP3 performs an essential role in parasite egress acting upstream of SUB1 (Arastu-Kapur et al., 2008). Based on this study, it was hypothesized that DPAP3 acts as a maturase of secretory proteins involved in egress and invasion. Recently, using a combination of biochemical and conditional knockdown approach, it has been proved that DPAP3 is an active protease residing in apical secretory organelles and its activity is critical for efficient RBC invasion and not parasite egress (Ghosh et al., 2018; Lehmann et al., 2018; Figure 2). The block in egress phenotype previously observed for DPAP3 inhibitors was due to off target or toxicity studies. Therefore, inhibitors targeting DPAP3 could be useful in disrupting the invasion pathway of malaria parasites. Several other reports have also suggested DPAP1 and DPAP3 as targets for malaria treatment and also blocking malaria transmission (Deu et al., 2010; Tanaka et al., 2013).

SERA proteins share significant homology with papain-like (Clan CA, family C1) cysteine peptidases and therefore speculated to have peptidase function. SERA 6, 7, and 8 possess canonical cysteine residue at its active site where as in SERA 5 this residue is replaced by a serine (Arisue et al., 2011). SERA 5 and 6 are relatively abundant soluble proteins localized to the PV and are known to be essential for development of blood stages of the parasite (Miller et al., 2002; Thomas et al., 2018). Essential roles of SUB1 and SERA6 in a co-ordinated proteolytic cascade leading to disassembly of RBC cytoskeleton have been shown (Ruecker et al., 2012; Thomas et al., 2018; Figure 2). SERA 5 function have also been linked to egress owing to its extensive proteolytic processing by SUB1 protease at around the time of parasite egress (Li et al., 2002; Yeoh et al., 2007; Withers-Martinez et al., 2014). Collins et al. (2017) have shown that in SERA 5 knockout parasites, the vacuolar membrane and the RBC membrane do not rupture properly at appropriate timings and lead to defective egress. This obstructs the propagation of newly formed merozoites. However, the question whether SERA 5 performs an important enzymatic role in the parasite had been a matter of debate until recent finding that SERA 5 plays an indispensable but non-catalytic regulatory role in the blood stage of parasite life cycle (Stallmach et al., 2015). Therefore, molecular events underlying the malarial egress seem to be tightly regulated by protease cascades and thus could be prime targets for malaria infection intervention.

## Malaria Parasite Threonine Proteases and Neutral Aminopeptidases

In *P. falciparum* only two threonine peptidases have been described: the proteasome and *Pf*Hs1V known to be involved in plasmodial metabolism (Gille et al., 2003; Mordmüller et al., 2006; Tschan et al., 2010). Threonine peptidases are characterized by presence of a particular N-terminal threonine residue at its catalytic center. In the *Plasmodium* genome, the proteasome subunit proteins are encoded by 14 genes and expressed all along the cell cycle while *Pf*HslV is encoded by one gene and expressed in schizonts and merozoite stages only (Gille et al., 2003). It is stated that apart from *Plasmodium* no other organism had been found to have both proteasome and HslV genes. Suitability of plasmodial proteasome as a drug target has been assessed in many studies by using proteasome inhibitors both *in vitro* and *in vivo*. Many of them have highlighted important roles of threonine proteases in asexual as well as hepatic and sexual stages in humans and Anopheles vector (Tschan et al., 2011). Mordmüller et al. (2006) had shown that epoxomicin is a very selective inhibitor of the proteasome which targets the plasmodial proteasome resulting in to enrichment of ubiquitinated proteins and thereby killing the parasites effectively. Exploration of HslV as a drug target in *Plasmodium* is of great importance due to following reasons: (1) it has no known sequence analog in the human host and thus reduces the chance of drug-mediated inhibition of host enzymes; (2) HslV is highly conserved among distantly related species of *Plasmodium* so these drugs could be used to target various species of the parasite. High conservation of Hs1V indicates its important function in *Plasmodium* life cycle and therefore seems to be a promising drug target candidate.

Aminopeptidases are cytosolic exopeptidases, show optimum activity at neutral pH (7.4) and catalyze the cleavage of amino acids from the N-terminal end of proteins and peptides (Gavigan et al., 2001). They are also known as MAPs because mostly they are metalloenzymes which contain Zn^2+^ ion in their active site. *P. falciparum* genome encodes nine MAPs, out of which four are methionine aminopeptidases where as other five include: a prolyl iminopeptidase, a prolyl aminopeptidase, a transmembrane M1-family alanyl aminopeptidase (PfM1AAP), an aspartyl aminopeptidase and a M17-family leucyl aminopeptidase (PfM17LAP). LAPs are critical for generation of intracellular pool of leucine in order to obtain isoleucine from the extracellular environment (Martin and Kirk, 2007).

**FIGURE 6:**
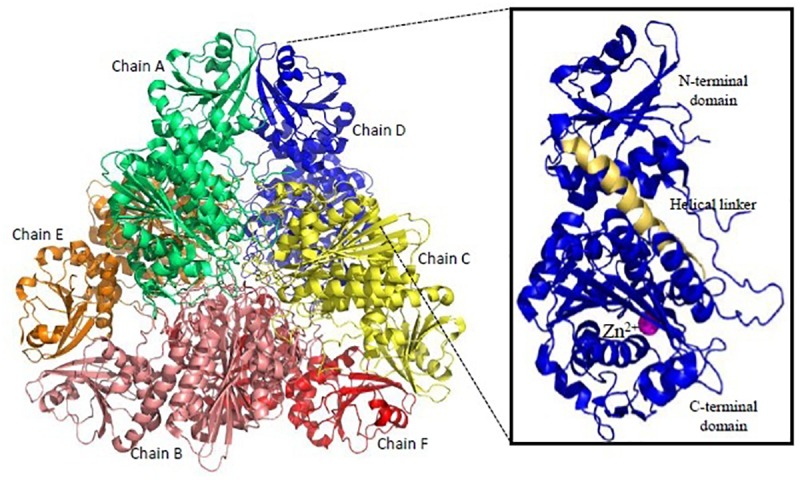
Overall structure of *Plasmodium* M17-family leucyl aminopeptidase (PfM17LAP). Cartoon representation of the biologically functional hexamer of PfM17LAP (PDB: 3LQX). Monomers are colored by chain: A (Green); B (Salmon pink); C (Yellow); D (Blue); E (Red); F (Orange). In inset is shown a monomer of PfM17LAP comprising of N and C-terminal domains connected with the helical linker (shown in pale yellow) and a Zn^2+^ ion (magenta). All structure figures have been made in Pymol.

Recombinant active proteins of PfM1AAP and PfM17LAP have been expressed, functionally characterized and crystal structures determined (McGowan et al., 2009, 2010). PfM17LAP adopts a hexameric arrangement of monomers for its physiological activity (Figure 6). Each monomer comprises an N-terminal regulatory domain connected by a ∼30 Å helix to the C-terminal catalytic domain (McGowan et al., 2010; Figure 6). Active site in each monomer is contained within the catalytic domain. Thus, the six active sites in the active hexamer from a central disk-like cavity which forms the entrance for the substrate to the enzyme interior and exit for the products. PfM1AAP has a single tightly bound Zn^2+^ ion in its active site whereas PfM17LAP has two metal-binding sites with one being an exchangeable site and the other a tight binding site. Removal of metal ion from the tight binding site results in an inactive enzyme (Skinner-Adams et al., 2010). Bestatin, a metalloprotease inhibitor derived from fungus *Streptomyces* inhibits both PfM1AAP and PfM17LAP by chelating their metal binding centers (McGowan et al., 2009; Yang et al., 2018). Inhibition of both these enzymes have shown lethal effects on *Plasmodium in vitro* and *in vivo* (Skinner-Adams et al., 2010). Therefore, inhibitors binding compactly and tightly to the active site and chelating the metal ion can show better anti-malarial effect. X-ray crystal structures of unbound PfM1AAP and rPfM1AAP in complex with inhibitor have revealed no major conformational changes occurring within the protease upon inhibitor binding (McGowan et al., 2009). Thus, targeting the active site access at entry (formed by domain IV, helical and C-terminal portions of the protease) and exit points (formed by domain I and IV) have been suggested for design of inhibitors. Novel inhibitors containing hydroxyamic acid zinc binding group and a variety of hydrophobic groups to probe S1’ pockets of Pf-M1, Pf-M17, Pv-M1, and Pv-M17 have shown potent inhibition of all the four molecular targets and high anti-malarial activity (Drinkwater et al., 2016; Vinh et al., 2018). Amino-benzosuberone derivatives have been proven to be highly selective inhibitors of Pf-M1, over Pf-M17 and shown high potency (Ki = 50 nM) in *in vitro* and *in vivo* assays (Bounaadja et al., 2017). “Malaria Box” screening efforts have led to identification of moderate inhibitors of Pf-M1 and Pf-M17 (Paiardini et al., 2015). Parasite and human host MAPs show (<35%) sequence identity and have different substrate specificities offering an exciting possibility for drug designing. Moreover, development of broad-spectrum anti-malarials could be possible because homologous aminopeptidases have been found in all four species of human malaria.

## Perspectives and Concluding Remarks

*Plasmodium* proteases are very well suited for target-based drug development approach because of their pathogen specific active site structures and enzymatic mechanisms and their involvement in a number of vital pathways which are essential for parasite survival. An overview of the roles of proteases involved in RBC invasion, Hb degradation and egress have been depicted in Figure 1, 2 and unique structural features have been collated in Table 1. A number of inhibitor-based studies have validated that blocking any of the involved proteases of the erythrocytic cycle can lead to quick drop in parasitemia and affect the malaria pathology significantly. Therefore, designing specific inhibitors against parasite proteases has been a feasible strategy for developing antimalarial drugs. However, the major limitations have been the presence of large protease families with functional redundancy and challenge in selectively targeting the active sites or unique structural domains of parasite-specific proteases. Most of the protease inhibitor research toward drug development was dependent on homology modeling and molecular docking approaches until elucidation of high resolution crystal structures of proteases over the years which played significant role in driving the inhibitor-design strategies.

**Table 1:** Biological functions and unique structural features of key *Plasmodium* proteases.

	Plasmodium
Protease family	protease(s)	Localization; function	Key structural features
Serine proteases	SUB 1	Stored in exonemes and released in to PV on rise of intracellular Ca^2+^; Egress and Invasion	N-terminal insertion in the prodomain forms a unique “belt” domain which also harbors a Ca^2+^ binding site unique to all *Plasmodium* orthologs; *Plasmodium-*specific insertion of ∼25 residues in the catalytic domain accommodating two Ca^2+^ ions
	SUB 2	Micronemes; invasion	Structure not determined
Aspartic proteases	Plm II	Digestive vacuole; cleaves native Hb	β-hairpin flap covering the binding cavity and flexible proline rich loop provide huge conformational flexibility at the binding cavity
	Plm V	Endoplasmic reticulum; processing of effector proteins for export to host cells	Presence of highly conserved unpaired cysteine, C140 in flap; presence of unique helix-turn-helix motif distinct from other plasmepsins
	Plm IX, X	Rhoptries and exoneme secretory vesicles; invasion and egress	Structures not determined
Cysteine proteases	FP-2, 2′, 3	Digestive vacuole; principally Hb degradation	N-terminal extension (12 aa) of the mature domain critical for folding; unique anti-parallel β-hairpin motif (14 aa) forming hemoglobin binding domain; Cys99-Cys119 disulfide bond is unique to falcipains unlike other papain-family proteases
	DPAPs	Apical secretory organelles; invasion	Structures not determined
	SERA 5, 6	Parasitophorous vacuole; breakdown of RBC cytoskeleton during egress	Structures not determined
Metalloaminopeptidases	M1AAP, M17LAP	Cytoplasm; protein catabolism	Central disk-like cavity formed by active sites in the hexamer; metal-binding sites (Zn^2+^) critical for activity


Hydroxyethylamine analogs have emerged as potential compounds used for targeting plasmepsins (I, II, IV) with effects being reciprocated in the *in vivo* models; however, the poor selectivity over its close human homolog, CatD and cross-reactivity among different Plms, yet remains a challenge toward development of any specific molecule as a drug. Now, better understanding of structurally distinct Plm V and multi-stage biochemical functions of Plm IX and Plm X have projected them as potential drug targets toward malaria eradication. The journey of FPs as attractive targets for drug discovery has been long since they are principal hemoglobinases. Although there is still lack of structural data on falcipain-2-Hb complex, the existing knowledge has highlighted the crucial role of unique anti-parallel β-hairpin, Hb binding motif in capturing its natural substrate. Further research is awaited on structure-guided design of inhibitors which interfere with falcipain-2-Hb complex formation. An advantageous approach would be designing broad-spectrum small molecule or peptide-based inhibitors targeted against one or more subfamily of Hb degrading enzymes like Plm inhibitors in combination with falcipain inhibitors. However, all these developments have to keep in to account the size of inhibitor for high oral bioavailability and reach to parasite-infected erythrocytes for desired efficacy. Events at invasion and egress being tightly regulated by activity of SUB proteases, present critical checkpoints which can be blocked. The “belt” loop and calcium-dependent regulation of SUB1 offer parasite-specific mechanism of action for the design of chemotherapeutic agents which can block the progression of parasite life cycle.

The success of any anti-malarial is determined by its specificity, i.e., the primary differences of the parasite target from the host. *Pf*Hs1V is a promising drug target as it is present only in pathogen and not in human host. However, the number of studies have been small and intensive research is needed in this direction. Nevertheless, *Plasmodium* proteases offer a set of unique “structural signatures” which can be used in designing very specific and potent inhibitors as anti-malarials. We conclude that an integrated approach including combination drugs against unique proteases of *Plasmodium* can be a way forward toward sustainable chemotherapeutic approach for fighting malaria.

## Author Contributions

SS evolved the theme of the article. MM and VS performed the literature survey and prepared the draft. All authors contributed in revision and finalizing the manuscript.

## Conflict of Interest Statement

The authors declare that the research was conducted in the absence of any commercial or financial relationships that could be construed as a potential conflict of interest.

## Abbreviations

AAP: alanyl aminopeptidase
ACT: artemisinin-combination therapies
DPAP: dipeptidyl aminopeptidase
DV: digestive vacuole
FPs: falcipains
HAP: Histoaspartic protease
Hb: hemoglobin
MAPs: metalloaminopeptidases
MSP: merozoite surface protein
LAP: Leucyl aminopeptidase
Pf: *Plasmodium falciparum*
Plm: plasmepsin
Pv: *Plasmodium vivax*
PV: parasitophorous vacuole
PVM: parasitophorous vacuolar membrane
RBC: red blood cells
RBCM: red blood cell membrane
SERA: serine-rich antigen
SUB: subtilisin-like protease
